# The Efficiency of Art-Based Interventions in Parental Training

**DOI:** 10.3389/fpsyg.2018.01495

**Published:** 2018-08-22

**Authors:** Liat Shamri Zeevi, Dafna Regev, Joseph Guttmann

**Affiliations:** ^1^The Graduate School of Creative Art Therapies, University of Haifa, Haifa, Israel; ^2^Art Therapy, The Academic College of Society and the Arts, Natanya, Israel; ^3^Faculty of Education, University of Haifa, Haifa, Israel

**Keywords:** parental training, art therapy, art based interventions, efficiency, quantitative research

## Abstract

In recent years, the field of art therapy has gained momentum, but art therapists still tend to work verbally during sessions with parents. The therapeutic approach presented here is anchored in the notion that the encounter between the art world and treatment creates a unique relationship between therapist, parents and the artwork. Eighty-seven parents of five to eight year olds filled in two quantitative questionnaires before and after a ten-month therapeutic intervention during which their child was treated through art therapy. Two other questionnaires were completed by the children and by the 14 art therapists. Three groups were tested: (1) Parental training with art-based interventions (intervention group). (2) Verbal parental training. (3) No Parental training. The parents in the first and second groups met the art therapist for parental training once every 3 to 4 weeks. In the intervention group the art intervention was based on a uniform protocol of exercises with various materials. It was hypothesized that a combination of art-based interventions during parental training (parents whose child was receiving art therapy) would contribute more to parent-child relationship, affect the parents’ self-perceptions of parental functioning, and improve the child's daily functioning than verbal parental training or no parental training, both in terms of the parents' and the child's perception. Analysis of the children's questionnaire indicated significantly higher scores in the intervention group than in the control groups for perceived cognitive abilities, perceived acceptance by peers and by the mother. Analysis of the parents' questionnaires indicated there was no difference in parental perceptions of their child, level of satisfaction, or efficiency between the intervention and the control groups. The art therapists reported improvement in the intervention group on almost every measure. When parents take part in a therapeutic experience that enables them to create and play with art materials, they may accept and appreciate their inner ‘child’ more easily. This may help them accept the fact that their own children are dependent on them, while at the same time acknowledging their need for autonomy, which can heighten children's perception of their own acceptance by peers and acceptance by their parents.

## Introduction

This study examined the efficiency of an innovative working approach in the field of parental training with art-based interventions. This approach is centered on creative work with the child's parents. It served to examine the parent-child relationship, in response to the growing need to integrate therapeutic work with parents in the field of art therapy.

Interest in understanding children's mental and developmental difficulties dates back to the earliest psychoanalytic approaches. Initially, psychoanalytic therapy for children made a clear separation between parents and children. Therapists emphasized the interpretation of unconscious conflicts and the strengthening of the ego (Freud, 1923, 1966) or the unconscious fantasies of the child (Klein, 1932). There was less focus on therapeutic work with parents as a factor that contributes to therapy. The Theory of Object Relations, which viewed the child's development process in terms of his/her relationships with other people and the environment, changed this orientation. Object relations theory considers the infant's initial relationship with the primary caregiver to be the basis for the development of the child's personality as an adult (Mahler, 1965; Winnicot, 1971; Ogden, 1990). However, alongside the integration of this approach, there was growing recognition of the importance of the parent as part of the child's therapeutic environment (Winnicott, 1964; Herzog, 2013). Over the years, numerous studies and observations have been conducted on the mother-child relationship, but father-child relations have rarely been examined. Studies show (e.g., Kochanska et al., 2007; Trowell and Etchegoyen, 2007) that the more involved the father is in raising his child, the more socially active the child will be, the less the pressure, and the less anxious the child will be. Therefore, recognizing the father's significant role in child development, this study dealt with the training of parents, fathers and mothers alike.

In addition to acknowledging the importance of the parental role in the child's emotional development (mother and father) in therapy (Fonagy et al., 2007; Barnet et al., 2008), a different conceptual approach to child therapy has emerged over the years. It has generated therapeutic models that do not center exclusively on the needs of the child, but also on the needs of the parent (Harel et al., 2010; Oren, 2015). Today, these models incorporate the parent into the therapeutic process in many ways, in the hope of prompting a change in the parent-child relationship.

Parental training is a broad field which is now considered to include many different aspects of the therapeutic intervention. It involves therapy with an adult client whose parental identity preoccupies him/her, or whose parental identity includes a form of distress that becomes the primary focus of therapy. This process functions on a continuum which may start from the provision of information on a specific subject, the provision of practical advice, or the clarification of specific issues as needed, and extends to therapy and training which enable parents to process past experiences that they project onto relationships with their children (Oren, 2012). Parental training can help parents deal with difficult feelings and foster understanding and acceptance while developing better conditions for adjustment and effective parenting. Sessions with parents are often complex because they provide a very specific space and time for dealing with a wide variety of needs and wishes. Generally, it is not easy for parents to allow an individual whom they do not know (the therapist) to have a close relationship with a child who is so dear to them, and parents often have an ambivalent attitude toward participating in their child's therapeutic process (Shamri-zeevi et al., 2015).

To better understand the importance of the parent's role in the child's emotional development, several approaches have been formulated over the years which deal with the needs of both the child and the parent. The approach utilized in the current study is based on parental training sessions for parents whose child is in art therapy. Children often need emotional therapy to deal with difficult issues or as a form of reinforcement. These include anxieties, fears, social difficulties, transitions and changes in life (for example starting first grade, residential relocation or parental divorce), anger management, developmental difficulties, depression, and others. In this approach, alongside therapy for the child, intermittent training sessions are held with the parents during which the therapist explains and describes the current status of therapy with the child (subject to confidentiality). The parents disclose and update the therapist about any issues at home and in the educational and social environments of the child. In the sessions with the parents, there is both training and a shared exploration of issues relating to parental behavior, while maintaining a clear and defined focus on the child. This training is part of a simultaneous therapeutic approach to the parent and child as defined by Chazan (2003) where therapy takes place separately for each individual (in separate sessions) by the same therapist. This approach posits that the therapist is the focal point between the parent and the child in transference and countertransference processes. Simultaneous therapy aims to connect up elements between parent and child, and is dependent on the therapist's ability to perceive the broad family structure when working with them, thus making interactions between the two individuals possible and heightening the understanding of the internal representations of both. This approach expands on the classical therapeutic view by examining the parallel processes and events that the parent and child experience with the same therapist (Nilsson, 2006).

Art-based parental training taps creative processes and the observation of the artwork as part of the parental training process (Deaver and Shiflett, 2011). Art therapy in itself is a therapeutic approach that has only developed in recent decades. The theoretical rationale of art therapy is that the creative process inherently has a therapeutic effect on the creator (Pratt, 2006). In the last 20 years, a broad theoretical and research foundation has been established based on the therapeutic potential of art therapy (see for example, Maujean et al., 2014; Schweizer et al., 2014) which rests on the idea that artistic expression does not only concern the end product, but that the process of creation and the end product together encourage significant mental processes.

The therapeutic approach in this study was based on the notion that the encounter between the world of art and the world of therapy creates a rich triangular relationship between the therapist, the client and the artwork. The presence of art materials in the therapy room provides parents with the opportunity to take part in a visual creative experience that utilizes their imagination and enables the symbolic and nonverbal expression of unconscious content (Schaverien, 2000; Case and Dalley, 2006).

Over the years, several authors have referred to the integration of art into parental training, specifically in parent-child art psychotherapy (Proulx, 2003; Lai, 2011; Buck et al., 2013, 2014; Regev and Snir, 2014). These authors argue that the use of creative materials may help parents overcome inhibitions and connect to unconscious memories, fears and wishes, and understand early conflicts that may be sources of difficulties in the relationship with their child through the language of art. In research on the integration of parental training and art therapy, a few studies have been conducted in the context of parent-child art psychotherapy in individual or group settings (Ponteri, 2001; Hosea, 2006; Plante and Berneche, 2008; Ya-hui et al., 2011; Pielech et al., 2013). Despite the small sample sizes, these studies all confirm the importance of art therapy with parents, and suggest that it affects the parent-child relationship and the child's self-perception positively. They also indicate that there is a significant need for parental training accompanied by therapeutic work with the child or with the parent-child dyad. However, no research to date has isolated the influence of art-based parental training as part of the art therapy process.

To respond to this need, the current study implemented quantitative research methods to examine the efficiency of an innovative working approach to art-based parental training interventions. Specifically, it examined whether the combination of art-based interventions during parental training (for parents whose child is receiving art therapy) would contribute more to the parent-child relationship, affect the parents' self-perception of parental functioning, and improve the child's daily functioning than verbal parental training (control group A) or no parental training (control group B).

It was posited that the intervention group (parental training with art-based interventions) would show a significant improvement in the outcome measures compared to control group A (verbal parental training) and control group B (no parental training) both in terms of the parent's and the child's perceptions. Four hypotheses were formulated:

1. The intervention group will show a significant improvement in the children's view of their self-perception following the intervention compared to the other groups.
2. The intervention group will show a significant improvement in parental perceptions of the relationship with their child following the intervention compared to the other groups.
3. The intervention group will show a significant improvement in parental perceptions of parental satisfaction and efficiency following the intervention compared to the other groups.
4. The intervention group will show a significant improvement in the evaluation of therapy outcomes by the art therapist in terms of the therapeutic bond, therapeutic openness/involvement, and overall evaluation of therapeutic outcomes following the intervention compared to the other groups.

## Method

This quantitative study included an intervention group and two control groups that were evaluated before and after a 10 month intervention program (pre-post design). The groups were assigned randomly, such that each participant had an equal probability of being placed in the intervention or control group. This helped ensure that there would be no systematic differences between the groups before treatment.

### Participants

The sample was composed of 87 families (children and their parents), aged five to eight (kindergarten to second grade) enrolled in the educational system and who were referred to art therapy by the counselor or the school psychologist for emotional and social difficulties, such as anxieties, fears, transitions and changes in life (for example starting first grade, residential relocation or parental divorce), developmental difficulties, behavioral problems and low self-esteem. The families were divided into three research groups: (1) 29 families, which comprised 33% of the sample, received parental training with art-based interventions (intervention group); (2) 30 families, which comprised 35% of the sample, received verbal parental training (without integrating art-based interventions, control group A); (3) 28 families, which comprised 32% of the sample, did not undergo parental training at all, apart from an initial familiarization session and a summary session with their child's art therapist (control group B). The study also included the 14 art therapists who treated these families, which was composed of 11 certified art therapists (with three to ten years of experience in the field) and 3 third year students from the School of Creative Arts Therapies at the University of Haifa, who were selected after being interviewed by the head researcher.

### Research instruments

The data were collected using five questionnaires which were completed by the children, parents and art therapists twice during the study, pre-treatment and after termination (pre-post design). All five questionnaires were translated into Hebrew, They were as follows:

#### Personal information questionnaire

The questionnaire was created specifically for this study and was completed at the beginning of the therapeutic intervention process. Parents were asked to provide personal information about the child and the family, and to answer an open question whether they were facing difficulties in their relationship with their child. In this study, the questionnaire was completed by one of the parents.

**PSPCSA** (pictorial scale of perceived competence and social acceptance for young children; Harter and Pike, 1984). This questionnaire examines the child's self-perception as perceived by the child. All of the items on the boys' questionnaire are identical to those on the girls' questionnaire. A high numerical value indicates high self-perception. The child's self-perception includes the constructs of perceived competence and perceived social acceptance, which are measured using a projective test composed of 24 items, which are divided into four scales: perceived cognitive abilities, perceived physical abilities, perceived acceptance by peers, and perceived acceptance by the mother. The responses are rated on a Likert scale ranging from 1 to 4 from “very true” for the child with the highest perception of ability (4) to “not at all” for the child with the lowest perception of ability (1). In the current study, the children completed the questionnaire with the help of the art therapist. The Cronbach's alpha for these subscales is reported to be ranged from 0.50 to 0.85, and the internal consistency for the entire questionnaire was 0.85 (Harter and Pike, 1984). In the current study, the Cronbach's alpha values as follows: perceived cognitive abilities α = 0.92, perceived physical abilities α = 0.81, perceived acceptance by peers α = 0.71, perceived acceptance by the mother α = 0.55, for a total score of α = 0.79.

**RFMQ** (relations with father/mother questionnaire; Mayseless et al., 1998). The original questionnaire was designed to assess the perceptions of teenagers, aged 13–17 regarding their relationships with their parents. Tal (2001) used the original questionnaire as a baseline but modified it to assess parents' perceptions of their relationships with younger children. The original questionnaire was made up of 63 items, whereas the later version has 55 items, divided into six scales: emotional intimacy, communication, reciprocity, control and supervision, open confrontation, and alienation and rejection. The responses are rated on a Likert scale from 1 to 6; a high score indicates that the parents assess their relationship with the child as positive. The Internal consistency in a repeated test for the original questionnaire was between 0.85 and 0.93 (Mayseless et al., 1998). The internal consistency for the adjusted questionnaire (Tal, 2001) was 0.74. In the present study, the questionnaire was completed by both parents; the Cronbach's alphas for the subscales were: emotional intimacy α = 0.83, communication α = 0.85, reciprocity α = 0.62, control and supervision α = 0.71, open confrontation α = 0.85, alienation and rejection α = 0.73, for a total score of α = 0.93.

**PSES** (parental satisfaction and efficacy scale; Johnston and Mash, 1989). This questionnaire, which assesses parental functioning, is composed of 17 items and is comprised of two factors: parental satisfaction and parental efficacy. Each factor has three components (parental satisfaction which covers degree of frustration, anxiety and motivation, and parental efficacy which covers degree of competence, problem solving skills, and parental resiliency).The responses are rated on a Likert scale from 1 (not at all) to 6 (strongly agree), with a high score indicating high parental functioning. Internal consistency for parental satisfaction was reported to be 0.75, and 0.76 for parental efficacy (Johnston and Mash, 1989). In the current study, the questionnaire was completed by both parents and the Cronbach's alpha for the subscales were: parental satisfaction α = 0.78, parental efficacy α = 0.79, for a general item reliability score of α = 0.84.

**TSR** (therapy session report—therapist's version; Kolden, 1993; Orlinsky and Howard, 1996). This self-report questionnaire was developed to examine the therapist's experiences and perceptions during therapy sessions and evaluates the client's progress in the short term. Handelzaltz-perry (2007) used the original questionnaire as a baseline but modified it to assess therapist's perceptions of each one of the therapy participants individually: mother, father and child. The original questionnaire was made up of 56 items divided into seven scales, whereas the later version was composed of 29 items, divided into three scales: therapeutic bond, therapeutic openness/involvement, and overall evaluations of treatment outcomes. Each item is scored on a Likert scale ranging from 1(not at all) to 5(strongly agree); responses are summed to obtain the overall score where a high score indicates high therapist evaluation. The internal consistency for the original questionnaire for therapeutic bond was reported to be 0.78, 0.74 for therapeutic openness/involvement (Kolden et al., 2000), and 0.80 evaluations of treatment outcomes (Kolden, 1991). In the current study, the questionnaire was completed by the art therapists and the Cronbach's alphas were α = 0.86 for the therapeutic bond (scale tested for each of the participants, mother α = 0.86; father α = 0.84, child α = 0.81), α = 0.84 for therapeutic openness /involvement (scale tested for each of the participants, mother α = 0.81; father α = 0.79, child α = 0.82), and α = 0.81 for evaluations of treatment outcomes.

## Procedure

The art therapists who worked with the children in this study met with the child's parents in a parallel process with the intervention group and with control group A. This process was composed of 8 to 11 individual sessions with the parents every 3 to 4 weeks, in a period of 10 months. The parents in control group B only met twice with the therapist, once for initial familiarization session at the commencement of their child's art therapy and a summary session once more at the end of the process. They did not receive parental training at all. The duration of each session with the parents was 50 to 60 min to allow for a meaningful conversation, artistic creation and the processing of the creative process (in the intervention group). For the intervention group, art-based interventions were combined with parental training in six to ten of the sessions. The therapists worked according to a standard protocol of exercises and suggestions for artmaking with various materials, which were consolidated into an “Arts-based Guide for Parental Training” prepared by the first author and other experts in the field. This guide included two main parts: the first part dealt with the parental training protocol based on the principles of the parent-child psychotherapy approach (Harel et al., 2010). This protocol was composed of recommended topics for discussion with the parents in the context of their relationship with their child. These included (1) Asking for a description of the difficulties the parents needed to cope with in their daily activities with their child; (2) Observation of the parents' communication with their child; (3) An examination of the parents' backgrounds in the context of their current behavior; (4) An examination of the patterns, thoughts, beliefs, and values that they transmit to their children; (5) An integration of the processes the parents experience in parental training and the child experiences in art therapy.

As part of the parental training process, there was collaborative thinking between the therapists and the parents regarding changes and their application in their day-to-day interactions with their child. This section also comprised the therapeutic protocol for art therapists for the control group A (verbal parental training). The second part of the guide was composed of 20 exercises and structured ideas for art-based therapeutic intervention techniques with parents so as to ensure as much similarity as possible. The exercises incorporated the use of different materials such as crayons, markers, colored pencils, gouache, clay, plasticine and collage work. The structured exercises had a number of sections such as how to arrange the setting, materials used in the exercise, detailed instructions for the therapist, and emphasis on talking points with the parents about the process and the artistic product. The exercises were compiled from 15 interviews with art therapists who use art-based intervention techniques in parental training (Shamri-zeevi et al., 2015), as well as the clinical experience of the first author.

The art therapy sessions with the children and the parental training sessions with the three groups were conducted by certified art therapists and third year students from the School of Creative Arts Therapies at the University of Haifa, with the provision of professional supervision from the University. The art therapists treated children from all three groups and were blind to the research objectives and questions.

## Ethics

Approval for this study was obtained from the Ethics Committee of the Faculty of Social Welfare and Health Sciences at the University of Haifa (Israel) and the Chief Scientist of the Ministry of Education, and protected the rights and privacy of all participants. Upon completion of the study the questionnaire identification key and the research reports were destroyed, leaving no identifying information about the participants in the results.

## Statistical analysis

To test for differences in demographic variables between groups, a Fishers' Exact Test p(X^2^) and a Bonferonni proportion test (see Table 1) were conducted. Table 1 lists the frequency of children in the sample according to gender and age for the three groups. When the effect was significant, this was taken into account when calculating the significant differences of the variable.

**Table 1:** Children's gender and age as a function of group.

		**Parental training with art-based interventions**	**Verbal parental training**	**No parental training**	**n**	p^*^
Child's gender	Male	41%	67%	54%	47	ns
	Female	59%	33%	46%	40	ns
	Total	29	30	28	87	0.051
Child's age	Five	21%	10%	39%	20	ns
	Six	28%	13%	29%	20	ns
	Seven	24%	37%	14%	22	ns
	Eight	28%	40%	18%	25	ns
	Total	29	30	28	87	0.046

* *p (Fishers' Exact Test) and Proportion Bonferonni*.

A series of preliminary analyses were conducted to examine whether the demographic variables had an effect on the dependent variables. For this purpose, one-way ANOVAs were conducted using multiple comparisons of the subgroups with Tukey's *post-hoc* analysis. These examined which variables should be included in the multivariate analysis (see Table 2).

**Table 2:** Summary ANOVA results for the significant effect of the different variables on the groups and on the research variables.

**Demographic differences**	**All research groups**	**PSPCSA (Total)**
Child's age	0.046	
City	<0.0001	
Mother's education	0.012	
Child's gender		<0.0001
Art therapist		<0.0001

## Findings

Means and standard deviations for the three groups' (intervention group and two control groups) outcome measures at pre-treatment and termination for all sub-scales of first to third hypothesis are provided in Table 3.

**Table 3:** Mean differences and standard deviations for the three groups for all sub-scales.

	**Parental training with art-based intervention**		**Verbal parental training**		**No parental training**	
	***M (SD)***	***N***	***M (SD)***	***N***	***M (SD)***	***N***
**SELF-PERCEPTION OF CHILD'S SUB-SCALES**						
Perceived cognitive abilities	1.39 (1.41)	58.00	1.67 (1.49)	58.00	1.41 (1.37)	56.00
Perceived physical abilities	0.85 (1.23)	58.00	1.59 (1.37)	58.00	1.15 (1.29)	56.00
Perceived acceptance by the mother	0.82 (0.98)	58.00	0.62 (0.89)	58.00	0.76 (0.99)	56.00
Perceived acceptance by peers	0.36 (0.94)	58.00	0.55 (1.12)	58.00	0.03 (0.99)	56.00
Aggregate variable of child's self-perception	0.85 (0.82)	58.00	1.11 (0.79)	58.00	0.84 (0.83)	56.00
**PARENT'S PERCEPTION OF THE RELATIONSHIP WITH THE CHILD SUB-SCALES**						
Emotional intimacy	−0.16 (0.77)	29.00	0.11 (0.54)	30.00	−0.01 (0.63)	28.00
Communication	−0.08 (0.61)	29.00	0.10 (0.88)	30.00	0.06 (0.75)	28.00
Reciprocity	−0.05 (0.46)	29.00	0.11 (0.66)	30.00	0.11 (0.88)	28.00
Control and Supervision	0.10 (0.59)	29.00	0.24 (0.65)	30.00	−0.04 (0.54)	28.00
Open confrontation	0.14 (0.44)	29.00	0.19 (0.72)	30.00	0.08 (0.53)	28.00
Alienation and Rejection	−0.08 (0.55)	29.00	0.00 (0.62)	30.00	0.22 (0.65)	28.00
Aggregate variable of parent's perception of the relationship with their child	−0.02 (0.44)	29.00	0.13 (0.37)	30.00	0.07 (0.43)	28.00
**PARENTAL SATISFACTION AND EFFICACY SUB-SCALES**						
Parental satisfaction	0.98 (0.55)	29.00	1.04 (0.62)	30.00	1.11 (0.63)	28.00
Parental efficacy	0.15 (0.44)	29.00	−0.02 (0.74)	30.00	0.15 (0.80)	28.00
Aggregate variable of parental satisfaction and efficacy	0.56(0.42)	29.00	0.51(0.52)	30.00	0.63(0.48)	28.00

The first hypothesis posited that in comparison to the two control groups, the intervention group would present with a significant improvement in the children's view of their self-perception. To examine the differences, a two-way MANOVA was performed to test the effect of art based parental training (independent variable) on the four self-perception sub-scales (dependent variables): perceived cognitive abilities, perceived physical abilities, perceived acceptance by peers and perceived acceptance by the mother.

We then examined whether there was a group effect on the sub-scales. As shown in Table 4, there was a significant multivariate effect of intervention group on the different sub-scales for self-perception [*Roy's Largest Root* = 0.118*; F*_(4, 165)_ = 4.88*, p* < 0.01, η^2^ = 0.106]. If significant differences were found for group on the sub-scales, the effect of group on each of the self-perception sub-scales was examined separately with a one-way ANOVA. A Levene's test was conducted to examine the homogeneity of the variance across groups for each of the dependent variables. Table 5 presents the results of the one-way ANOVA on self-perception for each sub-scale and the aggregate variable. There was a significant differences between groups for three of the four self-perception sub-scales: perceived cognitive abilities [*F*_(2, 167)_ = 2.24*, p* < 0.05], perceived acceptance by peers [*F*_(2, 167)_ = 4.34*, p* < 0.05] and perceived acceptance by the mother [*F*_(2, 167)_ = 2.62*, p* < 0.05]. However, no significant differences were found between groups on the perceived physical abilities sub-scale [*F*_(2, 167)_ = 0.47, *n.s*].

**Table 4:** Summary ANOVA results for all self-perception and social acceptance of the child sub-scales.

	**Roy's largest root**	***F***	***df* error**	***df***	***p***	**η^2^**
**ONE WAY**						
Constant	0.077	3.15	164	4	0.016^*^	0.071
Parental training with art-based intervention	0.118	4.88	165	4	0.001^**^	0.106
Child's gender	2.644	108.41	164	4	0.001^**^	0.726
Art therapist	0.027	1.09	164	4	0.361	0.026

* p < 0.05.

** *p < 0.01*.

**Table 5:** Summary one way ANOVA results of intervention group for self-perception of the child.

	***F***	***df* error**	***df***	***p***	**Levene's F**	**Levene's sig**.
Perceived cognitive abilities	2.24	167	2	0.035^*^	0.49	0.612
Perceived physical abilities	0.47	167	2	0.502	0.26	0.770
Perceived acceptance by the mother	2.62	167	2	0.028^*^	1.11	0.332
Perceived acceptance by peers	4.34	167	2	0.017^*^	3.76	0.025^*^
Aggregate variable of child's self-perception	0.43	167	2	0.204	0.09	0.913

* *P < 0.05*.

Given the significant differences between groups in terms of the child's perceived cognitive abilities, perceived acceptance by peers and perceived acceptance by the mother, follow-up analyses were conducted to identify the source of the differences between groups using Tukey's HSD. The results are presented in Table 6, and show a significant differences between the intervention group and control group A in terms of the child's perceived cognitive abilities [*M* = *0*.39, *SD* = 0.16, *p* < 0.05], the child's perceived acceptance by peers [*M* = 0.22, *SD* = 0.20, *p* < 0.05] and the child's perceived acceptance by the mother [*M* = 0.42, *SD* = 0.16, *p* < 0.05]. Thus scores were higher after integrating art-based interventions in parental training than in the verbal parental training group. The other differences between groups were not significant. Thus the first hypothesis was partially confirmed.

**Table 6:** Summary of follow-up analyses for significant perception of self-ability and social acceptance of the child subscales.

	**Parental training with art-based interventions**		**Verbal parental training**	
	**M(SE)**	***p***	**M(SE)**	***p***
Perceived cognitive abilities	0.39 (0.16)	0.037^*^	0.28 (0.15)	0.195
Perceived physical abilities	−0.18 (0.16)	0.759	−0.05 (0.16)	1.000
Perceived acceptance by the mother	0.42 (0.16)	0.027^*^	0.13 (0.16)	0.161
Perceived acceptance by peers	0.22 (0.20)	0.049^*^	0.13 (0.19)	0.160
Aggregate variable of child's self-perception	0.10 (0.10)	0.875	0.17 (0.10)	0.229

* *P < 0.05*.

The second hypothesis posited that the intervention group would exhibit significant improvement in the perception of the parent's relationship with the child. To examine the differences between the intervention group and the two control groups, a two-way MANOVA examined the effect of art based parental training (the independent variable) on six sub-scales of the parent's perception of the relationship with the child (dependent variables): emotional intimacy, communication, reciprocity, control and supervision, open confrontation and alienation and rejection. As shown in Table 7 there was no significant multivariate effect found in the intervention group between the different sub-scales examining the perception of the parents' relationship with their child [*Roy's Largest Root* = 0.091; *F*_(6, 77)_ = 1.17, *n.s*, η^2^ = 0.08]. The small effect of the therapeutic method suggests that changes in the therapeutic method most likely cannot account for the changes in the outcome of the parents' perception of the relationship with the child, but that other variables, aside from the therapeutic method, may explain these changes in the parents' perception of the relationship with the child.

**Table 7:** Summery variance of one way and two way MANOVA of the parents' perception of the relationship with their child.

	**Roy's largest root**	***F***	***df* error**	***df***	***p***	**η^2^**
**ONE WAY (BOTH PARENTS)**						
Parental training with art-based intervention	0.091	1.17	77	6	0.329	0.08
Child's age	0.111	1.40	76	6	0.223	0.10
Parent's education	0.027	0.34	76	6	0.912	0.02
City	0.067	0.85	76	6	0.533	0.06
**TWO WAY (THE IMPACT OF PARENT)**						
Parental training with art-based intervention	0.044	0.88	121	6	0.509	0.03
Parent	0.015	0.30	120	6	0.933	0.01

Although no significant differences were found on the different sub-scales, the effect of the groups on each of the sub-scales was examined separately by a one-way ANOVA. Levene's test was conducted first to examine the homogeneity of variance across groups for each of the dependent variables (see Table 8). This test showed that for each of the sub-scales representing the parents' perception of the relationship with the child, the variance was homogeneous across groups; hence a one-way ANOVA for each sub-scale met the statistical assumptions. Table 8 presents the results of this one-way ANOVA and indicates that there was no significant difference across groups for any of the sub-scales of the parents' perception of the relationship with the child, or in the aggregate variable. Therefore, the second hypothesis was not confirmed.

**Table 8:** Summary ANOVA results for all parent's perception of the relationship with the child sub-scales.

	***F***	***df* error**	***df***	***p***	**Levene's F**	**Levene's sig**.
Emotional intimacy	1.43	81	2	0.244	0.01	0.988
Communication	0.50	81	2	0.603	0.04	0.961
Reciprocity	0.38	81	2	0.685	0.85	0.429
Control and supervision	0.59	81	2	0.552	0.07	0.925
Open confrontation	0.04	81	2	0.959	2.63	0.078
Alienation and rejection	1.24	81	2	0.294	0.37	0.689
Aggregate variable of parent's perception of the relationship with their child	0.79	81	2	0.454	0.23	0.788

The third hypothesis posited that in comparison to the control groups, the intervention group would present with a significant improvement in parental satisfaction and efficacy following the art-based intervention in parental training. To examine the differences between the intervention group and the two control groups a two-way MANOVA was conducted on the two sub-scales of parental satisfaction and efficacy (dependent variables).

First we examined whether the magnitude of the group effect differed across subscales for parental satisfaction and efficacy (and in relation to the aggregate variable).

As shown in Table 9 there was no significant multivariate effect found for the intervention group between the different sub-scales of parental satisfaction and efficacy [*Roy's Largest Root* = 0.008; *F*_(2, 81)_ = 0.30*, n.s*, η^2^ = 0.00].

**Table 9:** Summery variance of one way and two way MANOVA of parental satisfaction and efficacy.

	**Roy's largest root**	***F***	***df* error**	***df***	***p***	**η^2^**
**ONE WAY (BOTH PARENTS)**						
Parental training with art-based intervention	0.008	0.30	81	2	0.738	0.00
Child's age	0.018	0.71	80	2	0.495	0.01
Parent's education	0.032	1.29	80	2	0.281	0.03
City	0.009	0.36	80	2	0.694	0.00
**TWO WAY (THE IMPACT OF PARENT)**						
Parental training with art-based intervention	0.016	0.97	248	4	0.379	0.01
Parent	0.003	0.20	123	2	0.818	0.00

Even though no significant differences were found for the effect of the manipulation on the different sub-scales, the effect of the groups on each parental satisfaction and efficacy sub-group was examined separately using a one-way ANOVA. For this purpose, a Levene's test was conducted first to examine the homogeneity of variance across groups for each of the dependent variables (see Table 10) which showed homogeneity of variance for each parental satisfaction and efficacy sub-scale indicating that a one-way ANOVA for each sub-scale met statistical assumptions. Table 10 presents the results of the one-way analysis of variance. No significant differences were found between groups in the sub-scales or in the aggregate variable. Therefore the third hypothesis was not confirmed.

**Table 10:** Summary ANOVA results for all parental satisfaction and efficacy sub-scales.

	***F***	***df* error**	***df***	***P***	**Levene's F**	**Levene's sig**.
Parental satisfaction	0.12	81	2	0.883	0.01	0.989
Parental efficacy	0.26	81	2	0.769	2.26	0.111
Aggregate variable of parental satisfaction and efficacy	0.29	81	2	0.749	0.57	0.567

The fourth hypothesis posited that the art-based parental training group would show significant improvement as evaluated by the art therapists in terms of therapeutic bond, therapeutic openness/involvement, and in the overall evaluation of the therapeutic outcomes following the intervention, in comparison to the control groups. To test this hypothesis, a one-way ANOVA with a Tukey's *post-hoc* follow-up analysis was used to compare the means of the intervention group and the two control groups. As shown in Table 11, no differences were found between control group A (verbal parental training) and control group B (no parental training). A significant difference was observed between the intervention group and the control groups for the three sub-scales of the therapeutic evaluation related to the mother, father, and child, as reported by the art therapists.

**Table 11:** Analysis of variance with Tukey's *post hoc* of the three groups to examine the effect of therapy as perceived by the art therapists.

	**Reasearch Groups**	**Mother**				**Father**				**Child**			
		**N**	**Mean**	**SD**	**Mean**	**N**	**Mean**	**SD**	**Mean**	**N**	**Mean**	**SD**	**Mean**
Therapeutic bond	Parental training with art-based interventions	29	3.84	0.49	0.03^*^	17	3.80	0.42	0.01^*^	29	4.16	0.63	0.00^*^
	Verbal parental training	30	3.54	0.54		14	3.37	0.50		30	3.47	0.76	
	Total	59	3.69	0.54		31	3.61	0.50		28	2.57	0.69	
	Fixed			0.52				0.46		87	3.41	0.94	
Therapeutic openness/involvement	Parental training with art-based interventions	29	3.74	0.84	0.48	17	3.56	0.86	0.050	29	3.45	0.63	0.00^*^
	Verbal parental training	30	3.60	0.66		14	3.07	0.65		30	3.08	0.68	
	Total	59	3.67	0.75		31	3.34	0.80		28	2.25	0.83	
	Fixed			0.76				0.77		87	2.94	0.87	
	Random											0.72	
	Random											0.70	
Overall evaluation of therapeutic outcomes	Parental training with art-based interventions	29	4.14	0.79	0.02^*^	17	3.88	0.78	0.49	29	4.99	0.98	0.00^*^
	Verbal parental training	30	3.63	0.93		14	3.64	1.15		30	3.93	0.98	
	Total	59	3.88	0.89		31	3.77	0.96		28	2.68	0.72	
	Fixed			0.86				0.96		87	3.75	1.19	
	Random											0.93	

p < 0.05*.

The findings indicated that art therapists' perceived a significant improvement in the intervention group in terms of the children's therapeutic openness/involvement (*M* = 3.45, *SD* = 0.63, *p* < 0.001) in comparison to control group A. There was a marginally significant improvement [*M* = 3.56, *SD* = 0.86, *p* < 0.1] in the therapists' perceptions in terms of therapeutic openness/involvement of the father by comparison to control group A. In terms of the therapeutic bond there was a significant improvement in the intervention group in terms of the art therapists' perception of the mother [*M* = 3.84, *SD* = 0.49, *p* < 0.05], the father [*M* = 3.80, *SD* = 0.42, *p* < 0.05] and the child [*M* = 4.16, *SD* = 0.63, *p* < 0.001] in comparison to control group A. In terms of the overall evaluation of therapeutic outcomes, the art therapists' perceived improvements in the mother [*M* = 4.14, *SD* = 0.79, *p* < 0.05] and the child [*M* = 4.99, *SD* = 0.98, *p* < 0.001] in comparison to control group A. Hence, the results of the ANOVA analysis and the Tukey's *post-hoc* analysis suggest that the fourth hypothesis was confirmed.

## Discussion

The aim of this study was to examine the efficiency of an innovative working approach in the field of parental training that integrates art-based interventions. The objective was to better understand the relationship between the art-based therapeutic process and its results. Specifically this study explored whether the integration of an art-based intervention in parental training (with parents whose child was in art therapy) would contribute to the parent-child relationship, affect the parents' self-perception in terms of their parental functioning and improve the child's daily functioning.

The hypotheses dealt with the differences between the intervention group (art-based parental training) and the two control groups (verbal parental training—control group A, and no parental training at all—control group B). Overall the prediction was that the intervention group would show better results than the control groups on all indices.

### The child's self-perception

The first hypothesis was partially confirmed. Three of the four sub-scales (perceived cognitive abilities, perceived acceptance by peers and perceived acceptance by the mother) increased significantly in the intervention group compared to the control groups. However, no significant differences were found between the groups in the sub-scale that examined the child's self-perception of physical abilities.

The term ‘self-perception’ is defined in the professional literature (Jacobs et al., 2003) as the sum of all attributes, abilities, attitudes and values that a person believes describe him or her. A significant function of self-perception in children is to set behavioral and motivational goals that are congruent with the way they perceive themselves, and to guide their social behavior and other activities (Harter, 1999). In several art therapy studies (Omizo and Omizo, 1989; Regev and Guttmann, 2005), mixed findings have been reported for 4 to 10 year olds' self-perceptions when integrating art therapy into group therapy, parent-child psychotherapy and individual therapy. Omizo and Omizo (1989) found that there was an improvement in self- perception in two out of four scales, whereas Regev and Guttmann (2005) observed no change in the self-perception of primary school-aged children.

Driessnack (2005) suggested that drawing makes it easier for children to communicate. In his overview, he found that children who are interviewed may respond more succinctly to questions as a result of their inability to retrieve information or understand a concept or event. By contrast, when children draw, they can generate new internal clues to events and thus organize the narrative in a way that makes it easier for them to communicate with their environment. It can be assumed that when parents draw, they connect to their children's experience which enabled them to perceive their children's abilities, which in turn may have had a positive effect on the children's self-perception in the intervention group. Gavron (2013) argued that artwork and the observation of the artwork both promote a process of “metaphorical insight” that makes art meaningful during parental sessions and allows for the acquisition of insight above and beyond describing and representing internal feelings and sensations. Even the use of creative materials on its own allows parents to be exposed to and access their own unconscious content which may enable them to adopt an additional conduit for observing themselves and their own children. When parents take part in a therapeutic experience that promotes creativity and play with art materials, they may allow themselves to connect, appreciate, and start to accept the “child core” within themselves, which at times has been forgotten or become a distant memory.

In this study, two indices that exhibited a significant increase in the intervention group concerning children's perceptions were the communication skills of the children with peers and with their mother, a finding that is consistent with theory and other studies (Oppenheim et al., 1997; Laible et al., 2004). These studies reported that the degree of warmth and emotional closeness that children feel when they see representations of the relationship with their parents is positively associated with adaptive behavior and good social ability with the peer group. Stern (2004) argued that self-esteem is based on an elementary level of awareness of self-processes that begins in infancy and is based on children's relationship with their mother and father figures. In this way, the mother produces a supportive framework for the development of the self in which the child feels valued and loved (Bretherton, 1990).

The hypothesis regarding the child's self-perception of physical abilities was disconfirmed. One possible explanation is that art therapy does not necessarily enhance or improve a child's physical abilities. The Regev et al. (2012) study of the effects of movement therapy on mother-child relationships and the child's self-perception found that in this kind of therapy, which emphasizes physical and motor skills, there was an improvement in the physical abilities perception index (in terms of measures before and after mother-child movement therapy interventions).

There was a significant increase in three out of the four self-perception indices in those children whose parents underwent art-based parental training sessions as compared to control group A. It should be noted that the children were the ones who received treatment and were the actual agents for measuring change. Only the children whose parents received art-based parental training reported that they sensed a change in their relationships with their parents, which led to an increase in the child's sense of self-worth. This may indicate that the parents, for their own reasons, were not yet willing to recognize the change in their child's behavior and relationship whereas the children reported a significant change. This is discussed in more detail below.

### Parents' perception of their children

The second and third hypotheses, which addressed the parents' perception of their children and the relationship between them, assumed that the intervention group would show a significant improvement in the indices measuring the perception of the parent-child relationship, parental satisfaction and efficacy following the intervention compared to the two control groups. These hypotheses were not confirmed.

There are several possible explanations for these findings. The first relates to time. In this study, the intervention took place over a period of 10 months on average. Although for children this may be a reasonable period of time to create a change in self-perception (in terms of perceived cognitive abilities, perceived acceptance by peers and by the mother, as can be seen in the first hypothesis), this may not be sufficient to bring about profound conceptual and practical changes in the parents' perception of their relationship with the child and their own self-perception (Toren and Shechtman, 2010). Within the intervention group, there was an increase in terms of the level of awareness of the difficulties and problems faced by the parents in their relationships with their children, as compared to the control groups. This suggests more time was needed for the parents to process the content that arose during the sessions than allocated in this study.

In addition, although the therapy period in this study was relatively short, the intervention protocol with the parents was not defined as a short-term dynamic therapy protocol, and therefore was not implemented as such. Short-term dynamic therapy (BDP—brief dynamic psychotherapy, or STPP—short term psychodynamic psychotherapy) is based on the principles of the psychoanalytic approach (Molons, 1998). Mann and Goldman (1982) presented a model based on the psychoanalytic approach to the concept of both realistic time and symbolic time. In this approach a central issue is selected for the limited time frame of the therapy. In outcomes research (some of which are comparative studies) conducted in the short-term dynamic approach, therapy has dealt with parental training, children with behavioral difficulties, and children with depression (Tsiantis et al., 2005; Trowell and Miles, 2011; Enebrink et al., 2015). The findings indicate that this type of therapy with these populations is effective. Enebrink et al. (2015), who conducted a study on 104 families, reported that a training process of only four sessions with the parents, over a period of 4 months, improved the parents' ability to show empathy toward their children, instill rules and boundaries, and increased parental efficacy and the well-being of the child.

In the current study parents met with the art therapist once every 3 to 4 weeks in the parental training groups (intervention group and control group A) and not as part of a standard therapeutic process in which the sessions take place once a week. Working according to the short-term dynamic approach with art-based parental training should be examined in further studies. Hence, the time frame of 10 months allotted in the study may have been too short and did not fully utilize the therapeutic protocol being examined. It is also possible that several extra sessions or the implementation of a short-term dynamic therapy approach would have resulted in greater perceived efficiency of parental training as assessed by the parents.

Parents may have had feelings of anger and frustration as the termination of the parental training process approached. This could have been experienced as forced termination and not welcomed at that stage. These feelings may have been expressed in the questionnaires they completed at the end of the parental training sessions. Forced termination was defined by Rosenfeld (1977) as a conclusion to therapy prompted by the therapist rather than by an improvement or progress in therapy or a decision by the client to leave. This definition emphasizes the unilateral nature of forced termination, and underscores the active role of the therapist in terminating the therapeutic relationship. Even though the final date of the therapeutic process was known and predetermined in the therapeutic/training process in this study, termination could still have been experienced by the parents as non-optimal and may have caused feelings such as insult, anger, abandonment and loneliness. Results from a number of studies (Fortune et al., 1992; Anthony and Pagano, 1998) indicate that the termination of the therapeutic process can trigger negative emotions in clients such as denial, anger, sadness, loss and anxiety, as well as positive feelings such as pride, a sense of accomplishment, maturity, and independence. However, when facing a forced termination, negative emotions will often appear more strongly. Keith (1966) coined the term “transfer syndrome” which refers to an increase in levels of anxiety experienced by the client when facing with the forced termination of the therapeutic process. He listed several symptoms that may make the process easier for the client, including downplaying the importance of therapy, its outcomes and the therapeutic relationship (Zuckerman and Mitchell, 2004).

### Evaluation of therapy outcome measures by the art therapists

The art therapists' therapy session reports also shed light on the advantages of parental training with art-based interventions and confirmed the fourth hypothesis. Namely, there was an improvement in the intervention group as compared to the two control groups for the indices in that the art therapists reported that in their opinion, the mothers, fathers, and children progressed on almost all scales. At the end of the process, the art therapists reported the efficiency of the training in a different and more positive manner than the parents. These results are consistent with studies that have examined the therapeutic relationship in which the therapist and the client were asked to evaluate the process and effectiveness of therapeutic outcomes (Manne et al., 2012; Holmqvist et al., 2016; Coyne et al., 2017). The therapists were more inclined to evaluate the therapy sessions in a more positive light than their clients. It should be noted that in this study the art therapists evaluated the therapy more positively than the parents despite being blind to the hypotheses. Furthermore, each art therapist treated families from all groups, which reinforces the validity of their reports. A combined outcome study that examined the perception of therapists and clients as to their expectations, the therapeutic relationship and the effectiveness of therapy found that the basis for differences in the assessment of therapy had to do with differences in their expectations and their interpretations of the components of the therapeutic process (Sewanee et al., 2017). For example, clients expressed a desire for more constructive therapy, they valued the therapist's support and validation during the sessions and were assisted by the therapists' suggestions and ideas. By contrast, the therapists who assessed the clients' desire to explore the therapeutic relationship wanted more time for therapy.

Second, the art therapists' perceptions of change the fathers underwent were positive, but to a lesser extent than for mothers and children. Studies have shown that fathers undergo less of a change in parental training (for example Tiano et al., 2013; Niec et al., 2015). In a study by Niec et al. (2015) composed of 120 mothers and fathers of children aged two to seven who coping with behavioral difficulties, fathers reported less readiness for change, a decreased sense of confidence in their ability to create a change in their relationship with their children, and perceived the parental training sessions as less important and effective than did the mothers.

## Conclusion and recommendations

Overall, the findings underscore the importance of art and creativity in children's emotional development. This study innovates by addressing the ways in which the relationship between parents and children in therapy can be improved by integrating art-based intervention techniques.

Future research should aim to develop focused, research-based approaches integrating art-based interventions into parental training sessions which can serve as significant tools for art therapists who work with children and their parents in the clinical field. Further development of this approach will enable art therapists to use creative tools in their work with parents much like when using them with children to enhance parents' reflective and empathic skills with their children.

The practical contribution of this research lies in its design of innovative directions and methods of treatment through art. It suggests a different approach to helping children with emotional difficulties who engage in art therapy. Whereas assistance is typically given to the children themselves, and sometimes verbal parental training is provided, this study attempted through a holistic approach, to view parents as creators so as to better understand the therapeutic process their children are undergoing, and to open a window onto emotional processes, experienced by parents of children with emotional difficulties. From a theoretical point of view, there have been no systematic studies examining models of art-based parental training. The results of this study have important practical implications. First, art therapists will be able to better determine the most appropriate methodology to work with parents. Second, art therapists can better gauge where to focus and deepen their relationship with parents, as a function of the child's difficulty. Third, art therapists will be better able to work in the clinical field by implementing the Art Based Parenting Guide that was written especially for this study. Fourth, other mental health professionals who do not use art as the primary instrument in their work may find art based interventions to be useful when working with parents.

This is the first time this type of data has been collected or presented in the form of a structured working model. Follow-up studies (as suggested above) should endeavor to encourage the development of focused, research-based models of integrating art-based interventions into parental training, which can serve as significant tools for therapists working with children and their parents through art in the clinical field.

This study has several limitations. A larger sample would enable a more thorough examination of the effect of combining art-based interventions with parental training sessions. Expanding the age range to the end of the latency period (age 12) would allow for a broader view of age-specific characteristics and parent-child relationship patterns. Finally, to define the contribution of art-based interventions in parental training, another avenue would be to lengthen the period of parental training to18 months. Assessing other protocols such as dynamic work in a short-term, goal-oriented psychodynamic approach would shed light on the ways in which a significant relationship between the therapists and the parents can be established. It may also allow for simultaneous termination of the research process with the end of the parental training therapeutic process. Another direction for further research would be to examine mother-child and father-child relationships separately. Such studies would enhance techniques of integrating art-based interventions in parental training.

## Author contributions

All authors listed have made a substantial, direct and intellectual contribution to the work, and approved it for publication.

### Conflict of interest statement

The authors declare that the research was conducted in the absence of any commercial or financial relationships that could be construed as a potential conflict of interest.
